# Postoperative Fungal Keratitis Managed by Anterior Chamber Washout and Intracameral Amphotericin-B: A Report of Two Cases

**DOI:** 10.7759/cureus.20769

**Published:** 2021-12-28

**Authors:** Anita Maniam, Lim Chee Min, Ling Kiet Phang, Francesca Martina Vendargon, Othmaliza Othman

**Affiliations:** 1 Ophthalmology, Universiti Kebangsaan Malaysia Medical Centre, Cheras, MYS; 2 Ophthalmology, Hospital Sultanah Aminah, Johor Bahru, MYS; 3 Ophthalmology, Universiti Kebangsaan Malaysia Medical Centre, Kuala Lumpur, MYS

**Keywords:** main corneal wound, phacoemulsification, vitrectomy cutter, anterior chamber washout, fungal keratitis

## Abstract

Keratomycosis is a significant cause of mono-ocular blindness, especially in tropical regions. Fungal keratitis developing in corneal incisions is very rare. We report the experience of treating two patients diagnosed with recalcitrant candida keratitis post-phacoemulsification with anterior chamber washout and deep debridement. The first patient was a 68-year-old woman who underwent left eye phacoemulsification nine months ago with a postoperative best corrected visual acuity of 6/6. The second patient was a 73-year-old man who had uneventful right eye phacoemulsification six months prior with a postoperative best corrected visual acuity of 6/9. Both patients used topical steroids postoperatively for more than three months and noted a drop in vision. Both patients had deep stromal infiltration and endothelial plaque at the primary corneal wound. They were unresponsive to topical, intracameral, and systemic antifungal therapy. Both patients underwent anterior chamber evacuation of hypopyon and endothelial plaque removal. Evacuation of hypopyon and removal of endothelial plaque was done with a 23G vitrectomy cutter using a low-powered vacuum controlled at 200 mmHg. The fluid inside the tubing was sent for culture analysis. We used viscoelastic coating on the endothelium to minimize the damage during the operations. Intracameral amphotericin B 15 µg/0.1 ml was given at the end of the operation. Postoperatively, both patients had clear corneas. The first patient’s visual acuity improved 6/18, and the second patient’s visual acuity improved to 6/9. Both cultures isolated *Candida parapsilosis* sensitive to amphotericin. These patient cases highlight that evacuation of the anterior chamber infiltration in recalcitrant fungal keratitis and intracameral injection of amphotericin B can be an effective adjuvant therapy.

## Introduction

Keratomycosis is one of the major causes of corneal blindness worldwide, with a reported incidence varying from 6% to 50%, but more prevalent in tropical regions than in the Western world [1,2]. The occurrence of fungal keratitis in a self-sealing phacoemulsification tunnel in which the infectious process started from a corneal incision is very rare. Fungi are less frequently reported as causative pathogens for phacoemulsification tunnel keratitis than bacteria, with *Aspergillus* species (spp.) the most common, followed by *Candida *spp., *Fusarium *spp., *Alternaria *spp., and *Scedosporium *spp. (only a few reported cases as of this writing) [3].

The management of fungal keratitis is challenging due to several factors. The anterior chamber inflammation in fungal keratitis can manifest as thick hypopyon or dense focal, fibrinous exudates adhered to the endothelium. Furthermore, in contrast with bacterial keratitis, hypopyon in keratomycosis generally contains fungal elements, and it is onerous to treat because of poor corneal penetration of topical antifungal agents [4,5]. Fungal keratitis caused by rapidly proliferating fungi can result in tissue necrosis and corneal perforation despite treatment with topic antifungal agents, oral antifungal, intracameral antifungal injection, or intrastromal antifungal injection. If opacity persists in the center of the cornea without perforation or when the fungal keratitis has a poor response to treatment, therapeutic penetrating keratoplasty is performed, and long-term treatment is needed [6]. Anterior chamber irrigation and evacuation of exudates have been reported as a treatment modality in recalcitrant cases of phacoemulsification tunnel keratitis without the need for therapeutic keratoplasty [7]. We present two phacoemulsification tunnel *Candida parapsilosis *keratitis cases, which were treated successfully with adjunctive anterior chamber evacuation of exudates.

## Case presentation

Case 1

The first case is a 68-year-old Malay woman with a history of diabetes, hypertension, and congestive heart failure monitored on regular follow-up. She underwent left eye phacoemulsification with posterior chamber intraocular lens implantation nine months ago at a private ophthalmologist clinic and had an uneventful recovery. Subsequently, she had left eye phacoemulsification at six months postoperatively and presented to a tertiary center reporting left eye pain, redness, and photophobia lasting two weeks. She had no history of eye trauma, foreign body presence, chemical injury, or contact lens usage. The vision of her left eye was 6/6. Upon anterior segment examination of the left eye, we noted mutton fat keratic precipitates with anterior chamber cells (2+). The intraocular pressure was 12 mmHg, which was within the reference range. On fundus examination, we noted her optic disc was pink with a cup disc ratio of 0.5, no sheathing of vessels, and no retinal scarring, retinitis, vitritis, vasculitis, or choroiditis. The findings from her systemic examination were unremarkable. Results from all other investigations, including results from her extensive uveitis workup, were unremarkable as well. She was diagnosed with persistent anterior uveitis and treated with prednisolone 1% eye drops administered every two hours and cyclopentolate 1% eye drops three times daily for her left eye.

Her left eye inflammation improved with decreasing anterior chamber cell activity from 2+ to occasional cells, so we tapered her prednisolone 1% to once daily for three months. Subsequently, the patient was referred to our eye center for logistical purposes. During her nine-month follow-up following her left eye phacoemulsification, her left eye vision dropped to 6/24. We noted corneal endothelial deposits with vascularization at the primary phacoemulsification wound, anterior chamber cells (3+), and occasional anterior vitreous cells. We noted no hypopyon, retrolental plaque, or vitritis. Considering the possibility of worsening keratitis, we halted the prednisolone 1% eye drops. We started her on levofloxacin 0.5% eye drops every two hours.

**Figure 1:**
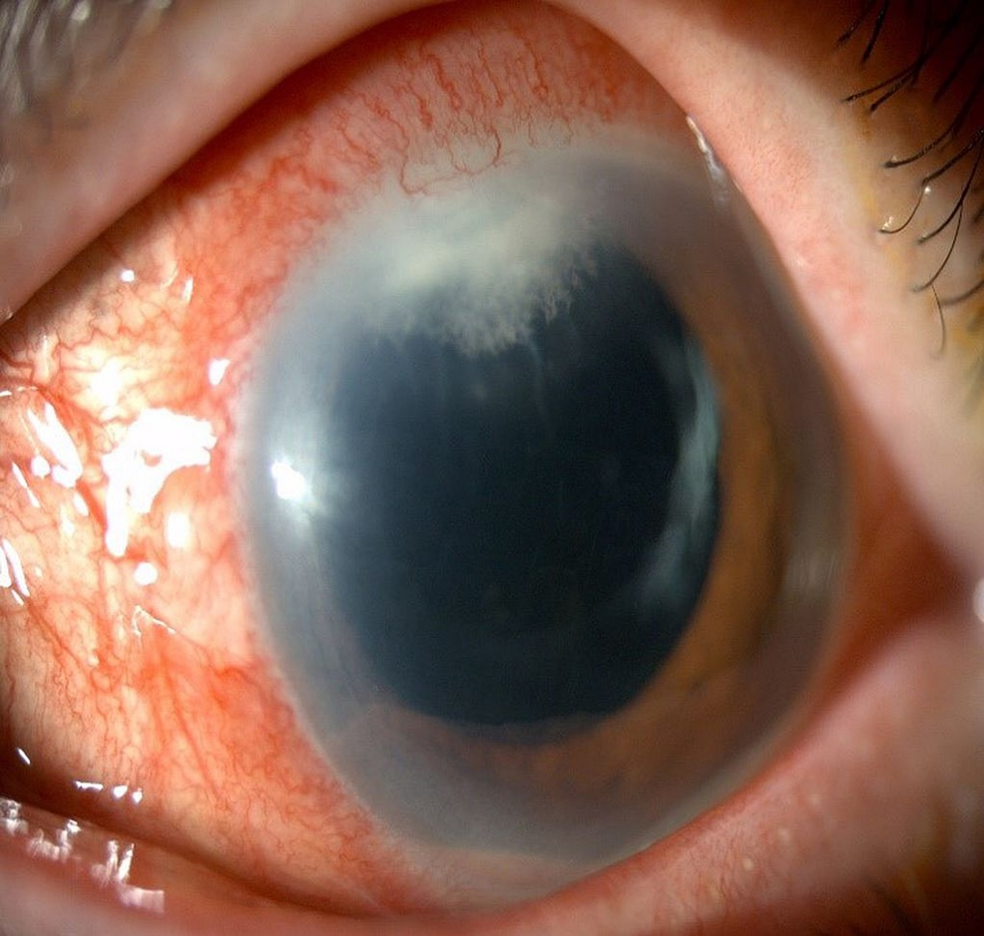
Anterior segment photo of left eye (Patient 1) showing phacoemulsification wound keratitis with fluffy infiltration measuring 2.5 mm (vertical) x 3.1 mm (horizontally) with a steak of hypopyon on the day of admission to our center.

**Figure 2:**
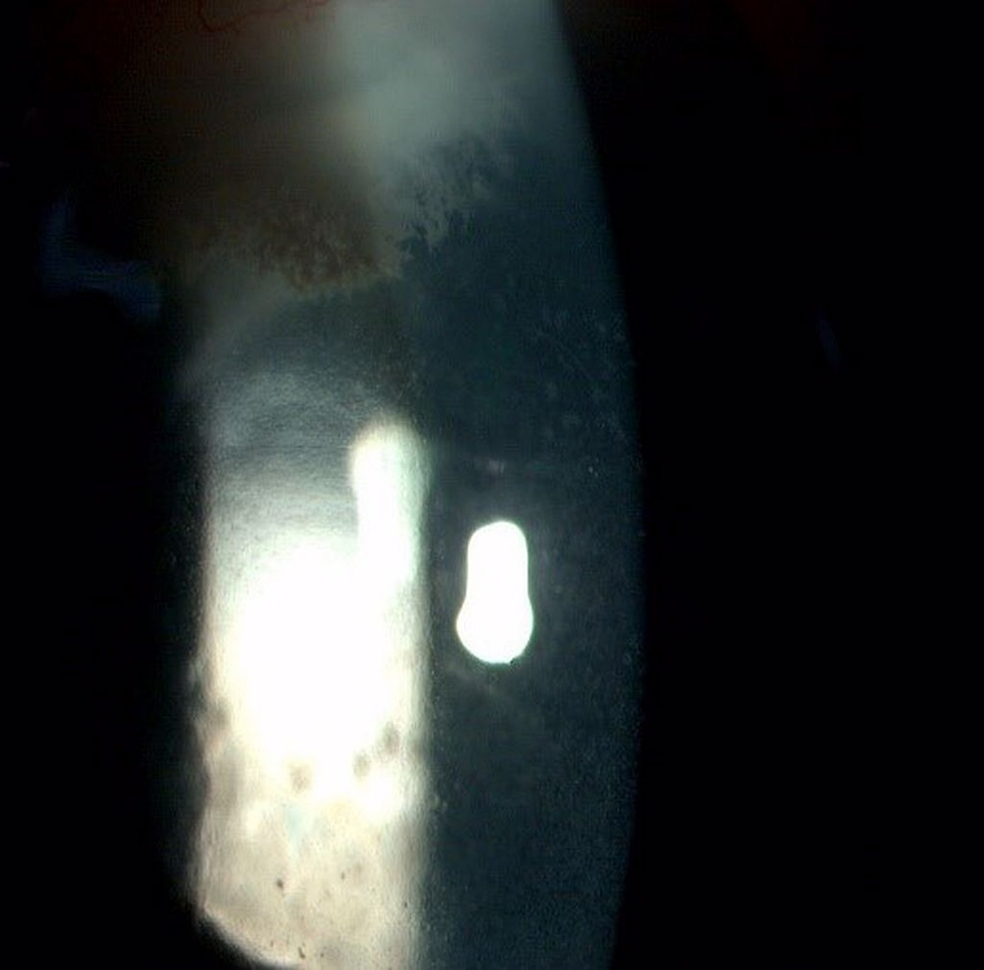
Slit anterior segment photo of left eye (Patient 1) showing stromal infiltration with feathery border, irregular margin, and fern-like pattern measuring 2.5 mm (vertical) x 3.1 mm (horizontally) temporal main wound tunnel on the day of admission to our center

**Figure 3:**
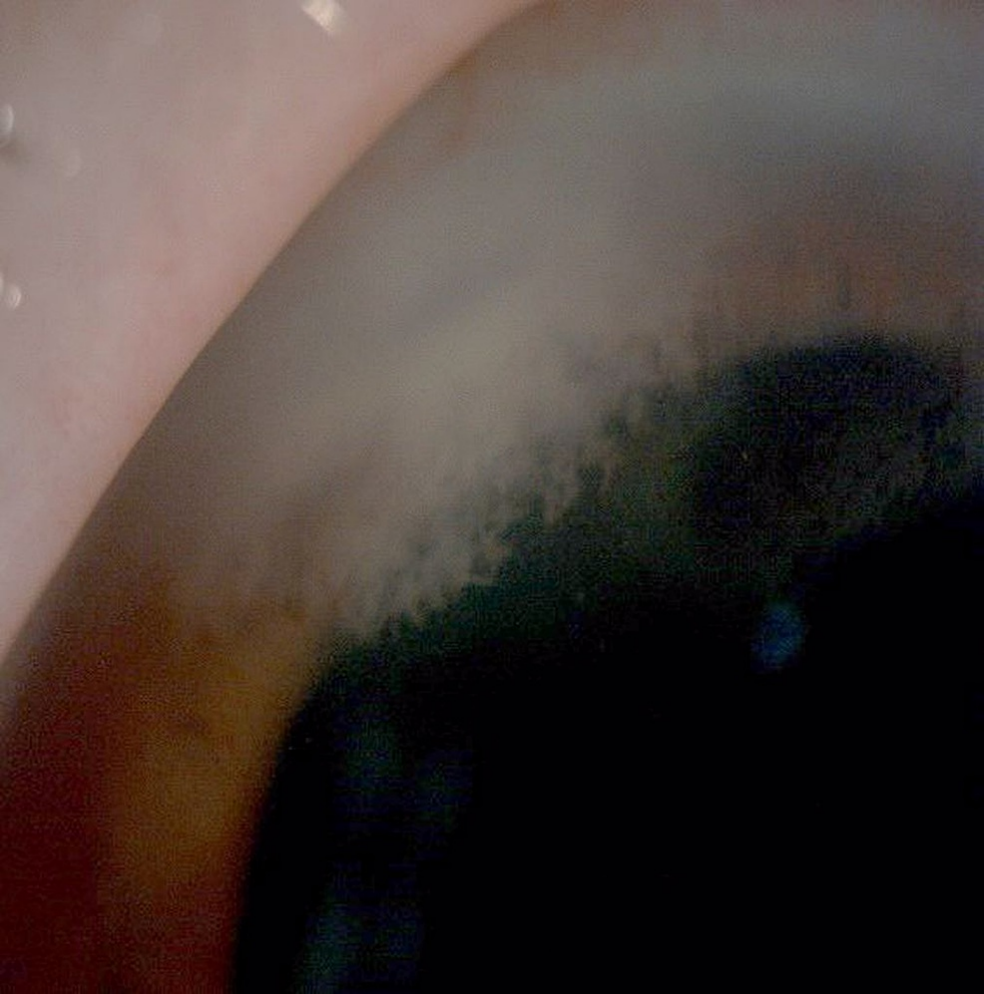
Anterior segment photo of left eye (Patient 1) showing the presence of stromal infiltration with feathery border, irregular margin, and fern-like pattern measuring 2.5 mm (vertical) x 3.1 mm (horizontally) temporal main wound tunnel on the day of admission to our center

The patient returned four days later, reporting concerns of left eye redness and swelling. Clinically, we noted worsening of her phacoemulsification wound keratitis with fluffy edged infiltrates measuring 2.5 mm (vertical) x 3.1 mm (horizontally) with a steak of hypopyon. We saw no vitritis, fungal ball, or loculation in the fundus (Figures 1-3). We diagnosed her with left eye phacoemulsification wound fungal keratitis. Samples from her left eye anterior chamber tapping were sent for culture and sensitivity analyses. She was admitted and started on fluconazole 0.2% eye drops hourly, amphotericin B 0.15% eye drops hourly, cefuroxime 5% eye drops hourly, gentamicin 0.9% eye drops hourly, atropine 1% eye drops three times daily over her left eye, and once-daily oral fluconazole 200 mg. Her left eye anterior chamber fluid culture and sensitivity analyses revealed *Candida parapsilosis *sensitive to amphotericin B and voriconazole. We administered intracameral amphotericin B 15 µg/0.1 ml injection every three days for a total of six injections.

**Figure 4:**
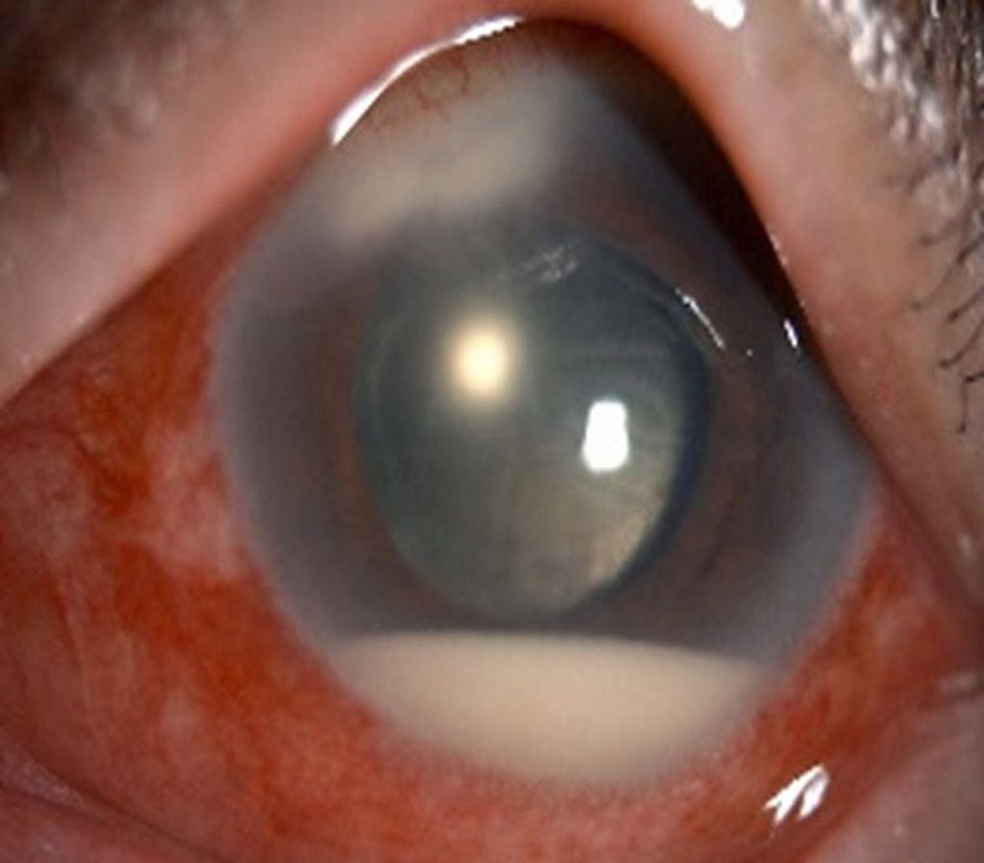
Anterior segment photo of left eye (Patient 1) showing worsening infiltration over main phacoemulsification wound and increasing level of hypopyon measuring 2.4 mm during the second week of admission

**Figure 5:**
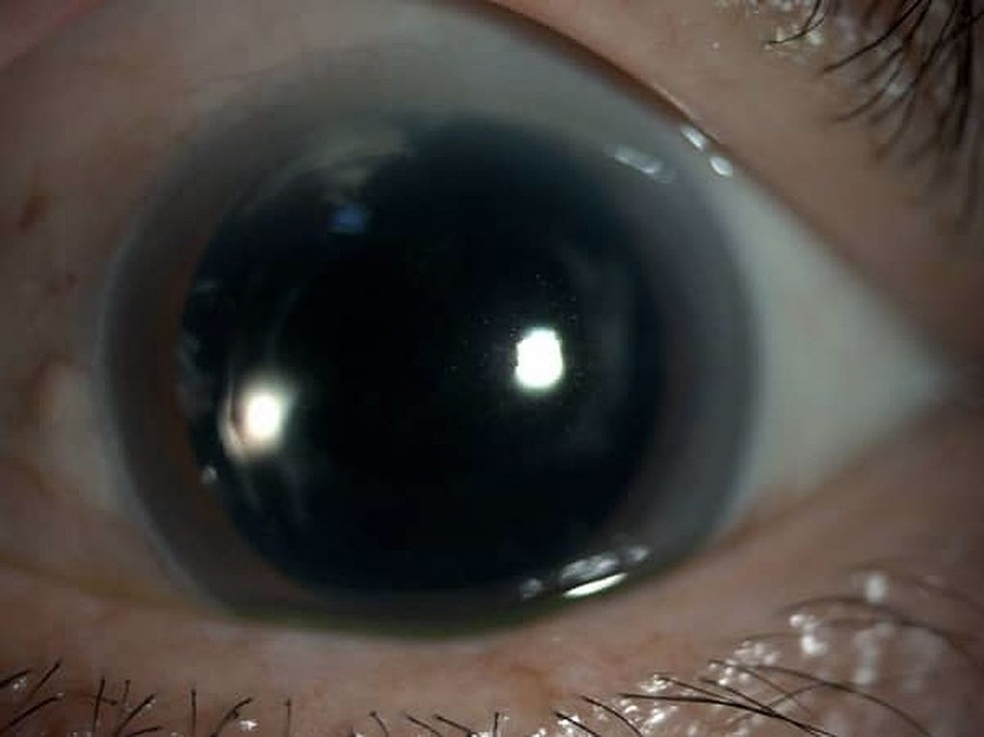
Anterior segment photo of left eye (Patient 1) showing clear central cornea, scarring at the phacoemulsification tunnel, and posterior chamber intraocular lens at one-month post anterior chamber washout and removal of endothelial plaque

During the second week of admission, the patient’s vision dropped to 6/60 with worsening infiltration over phacoemulsification of the primary wound and increased hypopyon measuring 2.4 mm (Figure 4). Subsequently, we replaced fluconazole 0.2% eye drops and daily oral fluconazole 200 mg daily with voriconazole 1% eye drops hourly, oral voriconazole 200 mg twice daily, and further intracameral voriconazole 100 µg/0.1ml three times with 48 hours; there were no adverse effects. At the third week of treatment, the patient underwent anterior chamber evacuation of hypopyon and endothelial plaque removal using a 23G vitrectomy cutter with a low-powered vacuum controlled at 200 mmHg using Stellaris Procedural Choice Vision Enhancement System (Bausch and Lomb Inc., Laval, Canada). Intracameral amphotericin B 15 µg/0.1 ml was given at the end of the operation. Postoperatively, the infiltration improved, and one month postoperatively, the patient had a clear cornea (Figure 5). Her vision improved to 6/18.

Case 2

A 73-year-old Chinese man with underlying treated stage two prostate cancer history with prostatectomy, radiotherapy, and remission for four years underwent uneventful right eye phacoemulsification with posterior chamber intraocular lens implantation at a district hospital six months prior to presentation with a best-corrected vision of 6/9 one month postoperatively. He was prescribed prolonged topical steroid postoperatively because of persistent inflammation: dexamethasone 0.1% eye drops for six weeks with tapering dose, then fluorometholone 0.1% eye drops with a tapering dose for another six weeks. He noticed a decline in his vision. At three months post phacoemulsification, his care team noted whitish matter at the phacoemulsification wound that could be cortical near the primary wound. His topical steroid dosage was further increased to prednisolone 1% eye drops every two hours [PME1] with moxifloxacin 0.5% eye drops every four hours . Upon review four weeks later, the patient developed keratic precipitate with anterior chamber cells (2+). The intraocular pressure was 16 mmHg (within the reference range). On fundus examination of the right eye, we noted the optic disc was pink with a cup disc ratio of 0.5, and there was no sheathing of vessels, retinal scarring, retinitis, vitritis, vasculitis, or choroiditis. Findings from his systemic examination were unremarkable. Considering the possibility of keratitis, we halted the prednisolone 1% eye drops. The results from all other investigations, including results from his extensive uveitis workup, were also unremarkable. His total prostate-specific antigen level was 0. 12 ng/mL, which is expected for a patient post-prostatectomy (reference range is 2.6 to 4.0 ng/ml). The patient presented again at six months after his right eye phacoemulsification. He reported pain in his right eye with blurred vision, photophobia, and redness lasting two weeks. He denied any history of eye trauma, contact lens usage, foreign body presence, chemical injury, or any further application of topical steroids since it was halted. Upon review, his right eye vision declined to a score of 6/24. We noted a large keratic precipitate with anterior chamber cells (3+) at the phacoemulsification main wound site. We noted no hypopyon. The findings from his fundus examination were normal, and he had no retrolental plaque, vitritis, chorditis, or vasculitis. At this point, his diagnosis was revised to right eye fungal keratitis with a differential diagnosis of worsening anterior chamber inflammation post topical steroid withdrawal. He was admitted for two weeks and started on moxifloxacin 0.5% eye drops hourly, amphotericin B 0.15% eye drops hourly, fluconazole 0.2% eye drops hourly, and atropine 1% eye drops daily over the right eye upon consulting the cornea team. However, given his worsening infiltration over the phacoemulsification main wound site, we stopped moxifloxacin 0.5% eye drops and started cefuroxime 5% eye drops hourly, and gentamicin 0.9% eye drops hourly over his right eye. We tapped his right eye anterior chamber and sent samples for gram stain, potassium hydroxide stain (to assess fungal hyphae), culture and sensitivity, and cytology workups. The culture result for anterior chamber tapping was negative. The cytology analysis revealed no malignant cells. Intracameral amphotericin B 15 µg/0.1ml was given once to the right eye.

**Figure 6:**
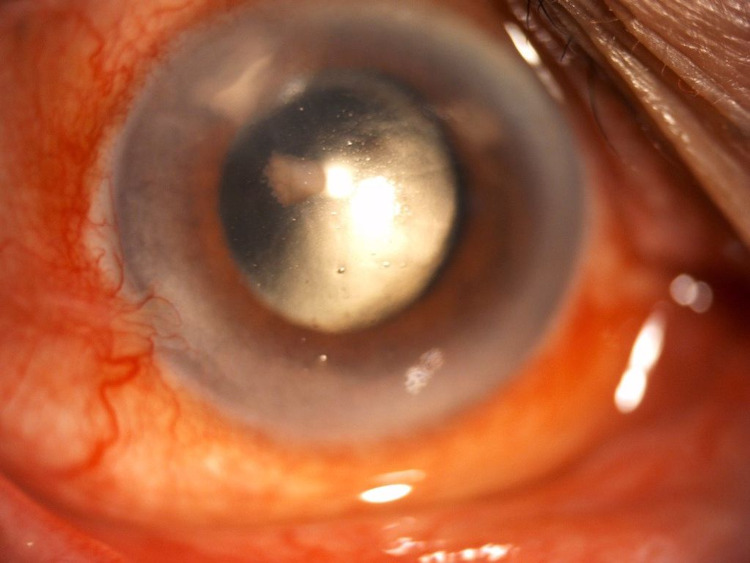
Anterior segment photo of right eye (Patient 2) with the presence of infiltration at main phacoemulsification wound measuring 1 mm (horizontal) x 2 mm (vertical) with another endothelial plaque with fluffy edges measuring 2 mm (horizontal) x 1 mm (vertical); endothelial plaque visible at main corneal wound with no hypopyon on the first day of admission to our center

**Figure 7:**
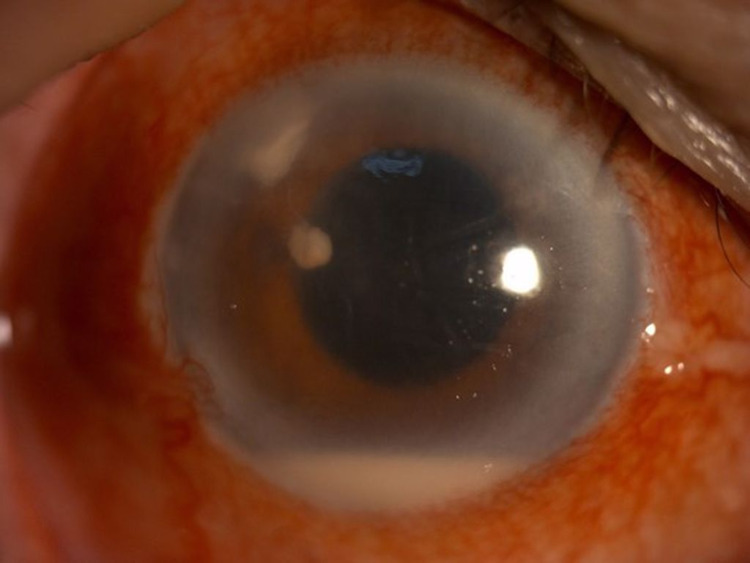
Anterior segment photo of the right eye (Patient 2) showing the presence of infiltration at main phacoemulsification wound measuring 1 mm (horizontal) x 2 mm (vertical) with another endothelial plaque with fluffy edges measuring 2 mm (horizontal) x 1 mm (vertical) with hypopyon measuring 1.6 mm at day nine of admission to our center

**Figure 8:**
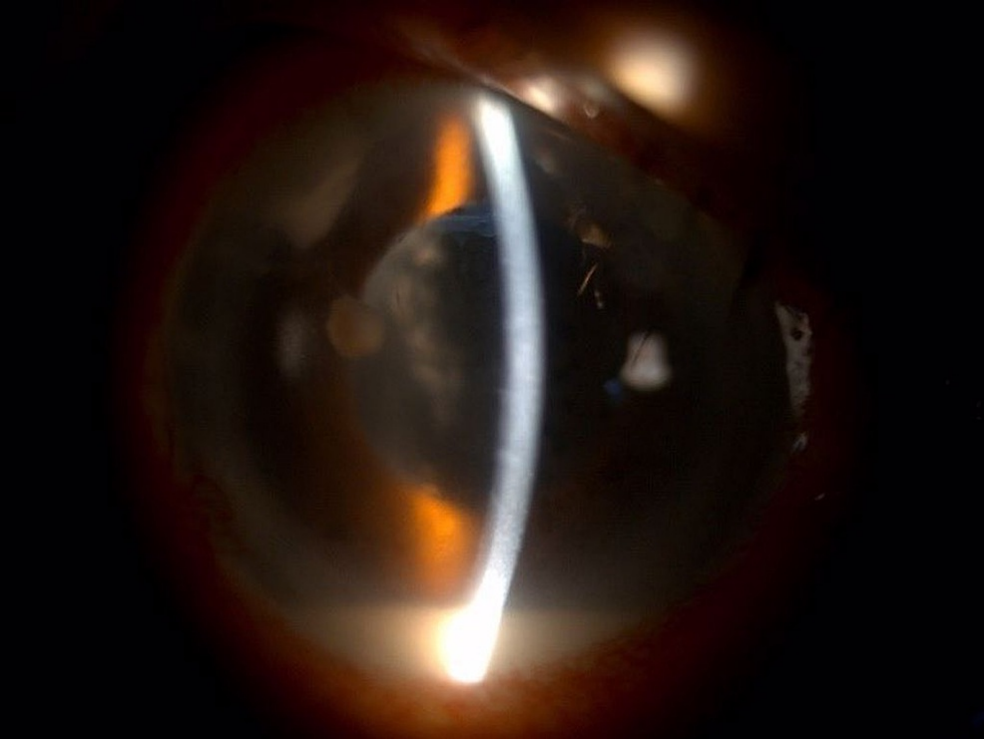
Slit anterior segment photo of right eye (Patient 2) showing the presence of infiltration at main phacoemulsification wound measuring 1 mm (horizontal) x 2 mm (vertical) with another endothelial plaque with fluffy edges measuring 2 mm (horizontal) x 1 mm (vertical) with hypopyon measuring 1.6 mm at day nine of admission to our center

**Figure 9:**
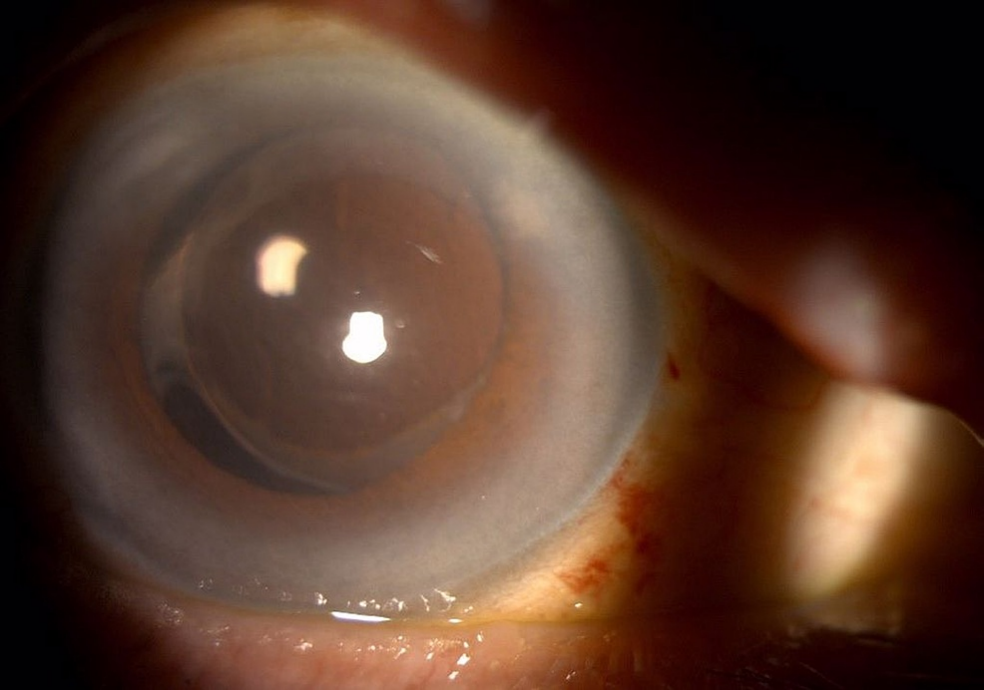
Anterior segment photo of the right eye (Patient 2) showing clear central cornea, scarring at the phacoemulsification tunnel, and posterior chamber intraocular lens at two weeks post anterior chamber washout and removal of endothelial plaque

Subsequently, the patient was transferred to our tertiary hospital for logistical reasons. Upon review, his right eye visual acuity was 3/60. We noted that his conjunctiva was injected with infiltration at the primary phacoemulsification wound measuring 1 mm (horizontal) x 2 mm (vertical) with another endothelial plaque with fluffy edges measuring 2 mm (horizontal) x 1 mm (vertical). There were anterior chamber cells (3+) with Descemet’s striae, no hypopyon, and his pupil was pharmacologically dilated with posterior chamber intraocular lens (Figure 6). The fundus examination revealed a pink optic disc with a cup disc ratio of 0.5 and a slightly hazy fundus view. However, there was no vitritis, vasculitis, or choroiditis over the right eye. B-scan ultrasonography of the right eye revealed a clear vitreous with a flat retina. His care team diagnosed him with right eye fungal keratitis post phacoemulsification. The anterior chamber tap for culture and sensitivity was repeated, but the results were negative. The patient was admitted to the ward and continued with amphotericin B 0.15% eye drops hourly, fluconazole 0.2% eye drops hourly, cefuroxime 5% eye drops hourly, gentamicin 0.9% eye drops hourly, and atropine 1% eye drops daily over the right eye. We started oral fluconazole 200 mg once daily, and we gave him intracameral amphotericin B 15 µg/0.1ml another three times at 72-hour intervals since admission. However, the patient’s condition was unresponsive to topical, intracameral amphoterin B and systemic antifungal therapy. The hypopyon worsened to 1.6 mm on day nine following admission (Figure 7 and Figure 8). We performed right eye anterior chamber washout and removal of endothelial plaque with a 23G vitrectomy cutter with a low-powered vacuum controlled at 200 mmHg using Stellaris Procedural Choice Vision Enhancement System (Bausch and Lomb Inc., Laval, Canada). The fluid inside the tubing was sent for culture and sensitivity, fungal polymerase chain reaction (PCR), and cytology analyses. Viscoelastic material was used to coat the endothelium to minimize the damage during surgery. Intracameral amphotericin B 15 µg/0.1 ml was administered at the end of the surgery. The culture isolated Candida parapsilosis sensitive to amphotericin. The fungal PCR test did not detect any fungal deoxyribonucleic acid, and the cytological analysis found no malignant cells. At two weeks postoperatively, the patient’s cornea was clear (Figure 9), and his vision improved to 6/9.

## Discussion

Fungal corneal tunnel infections after phacoemulsiﬁcation surgery are uncommon. Palioura et al. reported that there are 23 cases have been published in the literature [3]. Twelve eyes (52.2%) advanced to endophthalmitis with poor visual outcomes (visual acuity ≤ 6/120) in three patients, and loss of the eye occurred in four patients, three of which became phthisis bulbi and one underwent evisceration of the eye [3]. The latency period between the date of cataract surgery and the presentation of the fungal tunnel infection varied from three days to 15 years with a median of one month [3]. In this case report, the first patient had a latency period of nine months, and the second case had a latency period of six months prior to presentation. The causative pathogen for most cases were *Aspergillus* spp. (52.2%), two by *Fusarium* spp. (8.7%), two by *Alternaria* spp. (8.7%), three by *Candida albicans* (8.7%), one by *Candida parapsilosis* (4.3%), one by *Phialemonium curvatum* (4.3%), and one more by *Scedosporium apiospermum* (4.3%). In two reported cases, the fungal elements were detected on smear; however, the organisms did not grow on culture media [3]. Our first case isolated *Candida parapsilosis* from the anterior chamber fluid tap. However, in the second case, the anterior chamber fluid tap was negative twice. Subsequently, when anterior chamber washout and removal of endothelial plaque using a 23G vitrectomy cutter with a low-powered vacuum controlled at 200 mmHg was done in the second case, the anterior chamber washout fluid drained inside the sterile tubing intraoperatively was sent for culture and sensitivity and *Candida parapsilosis* was isolated sensitive to amphotericin B.

Phacoemulsification tunnel fungal keratitis is indeed a diagnostic and therapeutic challenge. Both patients underwent uneventful phacoemulsification via corneal wounds but later presented with fungal keratitis. Both patients were initially treated with prolonged topical steroids. Prolonged usage of topical steroids can worsen fungal keratitis and disturb the normal flora of the eye and local immunosuppression. The immunosuppression caused by topical corticosteroids is associated with the expansion of the infectious process in the corneal stroma and endothelium [8]. Numerous studies have concluded that the usage of topical steroids in the treatment of fungal keratitis resulted in detrimental effects and urged physicians to discontinue topical steroids in cases of suspected or culture-proven fungal keratitis [8]. Hence, topical steroids were discontinued in both our patients once fungal keratitis was suspected.

Even though antifungal agents such as polyenes, azoles, and fluorinated pyrimidines can be administered topically or systemically, they have disadvantages such as limited spectrum, poor penetration, limited clinical response, ocular surface toxicity, and prolonged course of treatment [9,10]. Intracameral and intracorneal amphotericin B have been reported to be beneficial in the management of deep fungal keratitis [11,12]. Since its discovery, voriconazole has been used extensively to treat ocular mycotic infections [13]. The use of topical and oral voriconazole in fungal tunnel infection have yielded good responses [14]. Some studies have also reported the benefits of using intrastromal voriconazole to treat recalcitrant fungal keratitis [15]. Both patients in this case report were given intracameral injections of amphotericin B. The first patient was also given an intracameral injection of voriconazole, given her poor response to the intracameral injection of amphotericin B. However, intraoperatively, upon the evacuation of hypopyon and removal of endothelial plaque, both patients received intracameral amphotericin B 15 µg/0.1 ml. Anterior chamber evacuation of hypopyon as a therapeutic modality combined with penetrating keratoplasty has been reported to be safe and effective [7,16]. Jain et al. found that evacuation of the anterior chamber exudates as an adjunct without penetrating keratoplasty in the treatment of patients of deep fungal keratitis with endothelial exudation was successful in achieving resolution of infiltration in eight of 23 cases (34.78%), and they concluded that patients who had deeper and larger infiltrates at presentation did not respond to the evacuation of anterior chamber exudates and eventually required keratoplasty [17]. Kitazawa et al. reported that three of their five patients had significant recovery after endothelial plaque aspiration with anterior chamber washout and another two patients needed therapeutic keratoplasty [7]. Both our cases were treated successfully with adjunctive anterior chamber evacuation of exudates. In both cases, we managed to prevent complications such as endophthalmitis and achieved good visual outcomes.

## Conclusions

The goal of treatment in postoperative fungal keratitis is to eradicate the infection, prevent endophthalmitis, and restore vision. Medical management alone with topical, intraocular, and systemic antifungals is often inadequate to control the infection in recalcitrant fungal keratitis. These two cases highlight that anterior chamber washout is an effective adjuvant therapy in managing postoperative fungal keratitis.
